# IMPACT OF NUMBER OF FUNCTIONAL TEETH ON INCIDENT MOBILITY DISABILITY IN OLDER ADULTS: FINDINGS FROM THE KFACS

**DOI:** 10.1093/geroni/igae098.4002

**Published:** 2024-12-31

**Authors:** Nahyun Lim, Hyung Eun Shin, Daehyun Lee, Heeeun Jung, Jae Young Jang, Hyunjin Cho, Chang Won Won, Miji Kim

**Affiliations:** Kyung Hee University, Seoul, Republic of Korea; Kyung Hee University, Seoul, Republic of Korea; Kyung Hee University, Seoul, Republic of Korea; Kyung Hee University, Seoul, Republic of Korea; Kyung Hee University, Seoul, Republic of Korea; Kyung Hee University, Seoul, Republic of Korea; Kyung Hee University, Seoul, Republic of Korea; Kyung Hee University, Seoul, Republic of Korea

## Abstract

The prevalence of mobility disability among older adults has a detrimental impact on their ability to live independently. It has been reported that losing functional teeth (FT) is linked to mobility disability. Nevertheless, there is insufficient longitudinal research investigating an association between the number of FT and incident mobility disability. We aimed to evaluate the association between the number of FT at baseline (2016–2017) and incident mobility disability after 6-year (2022-2023) in community-dwelling Korean older adults. A total of 1,090 older adults without mobility disability at baseline (50.2% women; mean age 75.3 ± 3.4 years) were analyzed from the Korean Frailty and Aging Cohort Study. FT were defined as total teeth, including remaining natural teeth and prosthetically restored teeth on panoramic radiography. Mobility disability was defined after 6-year as gait speed less than 1.0 m/s. The multivariate logistic regression analysis evaluated the association between FT and incident mobility disability. Participants having FT less than 20 were 26.3% (n=287), and the incident mobility disability was 30.2% (n=329). FT less than 20 were significantly associated with a higher risk of incident mobility disability in un-adjusted model (odds ratio [OR]: 2.27, 95% confidence interval [CI]: 1.71–3.00). After adjusting for confounders, FT less than 20 had a higher risk of incident mobility disability (OR: 1.95, 95% CI: 1.38–2.75). In conclusion, the number of FT was independently associated with incident mobility disability. Our findings suggest that maintaining FT at least 20 is essential to prevent mobility disability in older adults.

